# Nutrient comparisons of margarine/margarine-like products, butter blend products and butter in the US marketplace in 2020 post-FDA ban on partially hydrogenated oils

**DOI:** 10.1017/S1368980021004511

**Published:** 2022-05

**Authors:** Cecily Weber, Lisa Harnack, Abigail Johnson, Bhaskarani Jasthi, Janet Pettit, Jennifer Stevenson

**Affiliations:** 1 Division of Epidemiology and Community Health, School of Public Health, University of Minnesota, 1300 South 2nd St, Suite 300, Minneapolis, USA; 2 Nutrition Coordinating Center, School of Public Health, University of Minnesota, Minneapolis, USA

**Keywords:** Margarine, Butter, Partially hydrogenated oils, *Trans* fat

## Abstract

**Objective:**

To evaluate the fatty acid profiles and relevant vitamin and mineral compositions of margarine/margarine-like products and butter blend products available in the US marketplace and to compare with butter.

**Design:**

Analysis of the food and nutrient composition information available for margarine/margarine-like products, butter blend products and butter in the 2021 version of the University of Minnesota Nutrition Coordinating Center (NCC) Food and Nutrient Database.

**Setting:**

The US retail food marketplace in 2020.

**Participants:**

A selection of eighty-three margarine/margarine-like or butter blend products available in the USA in 2020 and regular and whipped butter (both salted and unsalted).

**Results:**

All products contained no or negligible amounts of *trans* fat. Mean daily values (DV) for SFA per 1 tablespoon ranged from 11 % for margarine/margarine-like tub and squeeze products to 18 % for margarine/margarine-like stick products and butter blend products. In contrast, one tablespoon butter provides 36 % of the DV for SFA. Results from ANOVA comparing the percent of total fat from SFA, PUFA and MUFA by product type indicated significant differences for SFA (*P* < 0·01) and PUFA (*P* < 0·01), but not MUFA (*P* = 0·07).

**Conclusions:**

Leading brands of margarine/margarine-like and butter blend products examined in this study were found to be in greater alignment with current dietary recommendations for fatty acids and cholesterol than butter. Margarine/margarine-like tub and squeeze products were found to be optimal over margarine/margarine-like stick products and butter blend products. Future research should include an examination of private label products.

In 2015, the US Food and Drug Administration (FDA) released its final determination declaring that partially hydrogenated oils (PHO) no longer maintain their status as Generally Regarded as Safe^(1)^. In addition, the determination states that PHO may no longer be included in food products, functioning as a ban on synthesised *trans* fat in the US food supply. The partial hydrogenation process (adding hydrogen to oils) allows for the creation of fats that are solid at room temperature, a quality that is seen as desirable for the development of many commercial food products such as margarine. However, during the process of partial hydrogenation, *trans* fatty acids are formed^(2)^. Synthesised *trans* fat has been shown to significantly increase LDL cholesterol^(3)^. Furthermore, observational studies indicate *trans* fat is associated with increased risk for CVD^(4)^.

Prior to the FDA ban on PHO, margarine and margarine-like products in the US marketplace were a source of *trans* fat due to the use of PHO in product formulations^(5–7)^. Due to the use of PHO and therefore *trans* fat in these products, dietary recommendations in the past have recommended minimising or avoiding the use of hard or stick margarines along with minimising intake of fats and oils high in saturated fat and cholesterol^(8)^. The *Dietary Guidelines for Americans 2020–2025* recommends that SFA intake does not exceed 10 % of daily energy intake and that cholesterol intake be as low as possible without compromising the nutritional adequacy of the diet^(9)^. Unsaturated fatty acids are recommended in place of SFA.

The FDA determination that PHO may no longer be used in food products led to the need for food manufacturers to carry out product reformulation for commercial products that included PHO. To allow time for manufacturers to carry out needed food product reformulations, a final compliance date of January 1, 2020 was established for all food products except those with petitioned uses. Foods manufacturers with petitioned uses of PHO had until January 1, 2021 comply^(1)^.

To our knowledge, no studies have reported on the fatty acid profile of margarines in the US marketplace post mandatory removal of PHO from foods, yet this information is crucial to registered dietitian nutritionists and public health professionals in providing dietary guidance. To address this information need, this study aimed to describe and compare the fatty acid profiles and relevant vitamin and mineral content of margarine/margarine-like products, butter blend products and butter found in the United States marketplace in 2020. It was hypothesised that post FDA ban on PHO, margarines/margarine-like and butter blend products no longer contain *trans* fatty acids and contain less saturated fat than butter, making them superior options to butter for CVD prevention.

## Materials and methods

### Data source

Data on the nutrient composition of butter, butter blends and margarine/margarine-like products available in the US marketplace in 2020 were obtained from the 2021 version of the University of Minnesota Nutrition Coordinating Center (NCC) Food and Nutrient Database (to be publicly released July 2021)^(10)^. This Database is maintained using a standard set of procedures that are described in detail elsewhere^(11–14)^.

When updating the nutrient content information for brand name margarine/margarine-like products and butter blends, a full listing of products available from major food companies is first assembled by NCC database scientists. Ingredient and Nutrition Facts label information is sought for each individual product. This information is typically obtained from food company websites or major online grocery retailer websites. Nutrient values for each product are then derived using a multifaceted approach. This approach includes assigning nutrient values as provided on the product’s Nutrition Facts label. For nutrients and food components not included on the label, nutrient composition data in the USDA National Nutrient Database Standard Reference is used if available. Values from other food and nutrient databases and articles in scientific journals containing values obtained using appropriate analytic methodologies may also be used. Imputation procedures are used for nutrients and components not available on the product label or in Standard Reference or other sources. Imputation procedures used include calculating values by creating product formulations using an NCC developed program^(15)^.

### Margarine/margarine-like products, butter blend products and butter in the NCC database and product selection

NCC aims to include all products available from food companies considered to be market leaders within a food product category, with the identification of leading companies based on publicly available industry reports (if available) and expertise of the NCC database scientists. To keep pace with marketplace changes, products and nutrient values for products in various food categories are updated on a rotating basis over time. During the update process, additional food companies are added as necessary to ensure leading companies are included.

In 2020, the margarine product category of the NCC database was updated, and these updates were included in the 2021 version of the NCC Food and Nutrient Database. The margarine category in the 2021 version of the Database included 83 products sold by 5 food companies (see Table 1). For the present study, the products were classified as margarine/margarine-like tub and squeeze products, margarine/margarine-like stick products and butter blend products. Butter blend products were considered to be those that contained oil and had cream as the first ingredient, contained cream as the second ingredient with water as the first ingredient or contained butter as an ingredient. These products were not separated into ‘tub and squeeze’ and ‘stick’ subcategories because all but two products were in the tub form.

**Table 1:** Number of margarine/margarine-like and butter blend products sold in the USA in 2020 by five food companies

	Margarine/margarine-like tub and squeeze products	Margarine/margarine-like stick products	Butter blend products	Total
Conagra brands, Inc.				
Blue Bonnett	3	3		6
Earth balance®	10	2		12
Fleischmann’s	3	2		5
Move over butter	1			1
Parkay	3	2		5
Smart balance	9	1	2	12
Land O’ Lakes, Inc.				
Land O’ Lakes	2	1	10	13
Olivio premium products Corp.				
Benecol®	2			2
Olivio	4			4
Richardson food & ingredients				
Canola harvest	1			1
Upfield				
Brummel and Brown	1			1
Country Crock®	6	5		11
I can’t believe it’s not butter!®	5	1		6
Imperial®	1	1		2
Pure blends	2			2
Total	53	18	12	83

The term ‘margarine/margarine-like’ products was used because most of the products did not appear to meet the FDA standard of identity for margarine, which specifies that 80 % of the product by weight must be fat^(16)^. Margarines must also contain a vitamin A content of 15 000 IU/pound or more and may optionally contain vitamin D not less than 15 000 IU/pound.

The NCC Food and Nutrient Database do not contain commercial brands of butter due to the standardised nature of butter and limited variation in nutrient composition between brands. The 2021 version of the Database contains nine variations of butter. Four variations were chosen as standards for comparison primarily based on their common use and availability in the marketplace. These include salted and unsalted regular butter and salted and unsalted whipped butter. Whipped options were included for their potential as lower calorie and lower saturated fat alternatives to regular butter.

### Selection of nutrients to analyse and food amount

Nutrients examined include energy (kcal), total fat (g), *trans* fatty acids (g), SFA (g), PUFA (g), MUFA (g), *n*-3 fatty acids (g), *n*-6 fatty acids (g), conjugated linoleic acid (g) and cholesterol (mg). Percent daily values (DV) were calculated for total fat, SFA, sodium, calcium, vitamin A, vitamin D and vitamin E. Daily values are defined by the FDA as reference amounts of nutrients to consume or not exceed each day. The % DV is the percentage of the DV for each nutrient in a serving of the food^(17)^. In addition, the proportion of total fat was examined for each of the major classes of fatty acids.

All nutrient values are reported per one tablespoon of the product. This amount was selected because it is the Reference Amount Customarily Consumed for butter and margarine^(18)^.

### Statistical analysis

Mean and SD values of nutrients for the product categories were calculated. Minimum and maximum nutrient values were also determined. All calculations were completed using Microsoft® Excel for Mac (version 16.48). Mean % DV were calculated by first dividing the nutrient value for each product by the DV for that nutrient^(17)^ and multiplying by 100, then summing all products’ calculated DV and dividing by the number of products. The numbers of products considered to be ‘good’ or ‘high’ sources of nutrients in accord with FDA labeling standards (10–19 % DV ‘good’ and >20 % DV ‘high’)^(19)^ were also examined. Percentages of total fat for the major classes of fatty acids were calculated by dividing each product value for the specified fat by the total fat in one serving (1 tablespoon) of that product and multiplying by 100. These values were then summed and divided by the number of products in the category to determine the mean value. Nutrient values and % DVs for the four comparison butter products are presented as individual values, not group means.

ANOVA analyses were conducted to determine whether the percent of total fat from each major class of fatty acids were significantly different between margarine/margarine-like tub and squeeze products, margarine/margarine-like stick products, butter blend products and butter. For this calculation, the four butter comparison products were combined into one category, ‘butter.’ A one-way ANOVA test was run for each fatty acid category, with a *P*-value < 0·05 considered statistically significant.

## Results

Table 2 presents the mean, sd, minimum and maximum values for energy, total fat, SFA, PUFA, MUFA and cholesterol for each product category. Individual values for the butter comparisons are also presented. For the margarine/margarine-like and butter blend categories, mean energy content ranged from 68 to 87 kcal per tablespoon while regular butter contained 102 kcal and whipped butter contained 68 kcal per tablespoon. For total fat, mean % DV ranged from 10 to 12 % between the margarine/margarine-like and butter blend products while regular butter contained 15 % DV and whipped butter contained 9 % DV. Tub and squeeze margarine/margarine-like products had the lowest % DV for SFA (11 %) while the butter blend and margarine/margarine-like stick products contained an average of 18 % DV. Regular and whipped butter contained 36 % and 21 % of the DV for SFA, respectively. On average, margarine/margarine-like and butter blend products contained at least twice the amount of PUFA per tablespoon (1·2–2·5 g) compared to the regular and whipped butters, which contained 0·4 g and 0·3 g, respectively. The mean % DV of cholesterol in the margarine/margarine-like products was 0 % with butter blend products containing 4 % and regular and whipped butters containing 10 % and 7 %, respectively. All of the margarine/margarine-like and butter blend products and butters contained negligible amounts (<0·50 g/1 tablespoon) of *trans* fat and conjugated linoleic acid (data not shown).

**Table 2:** Energy and fat content of one tablespoon of margarine/margarine-like products, butter blend products and various butters sold in the USA in 2020 by five food companies

	Mean/value	sd	Min	Max
Energy (kcal)				
Margarine/margarine-like tub and squeeze products	68	20	35	100
Margarine/margarine-like stick products	84	18	50	100
Butter blend products	87	17	50	100
Butter, salted	102			
Butter, unsalted	102			
Whipped butter, salted	68			
Whipped butter, unsalted	68			
Total fat (g)				
Margarine/margarine-like tub and squeeze products	7·5	2·3	4·0	11·0
Margarine/margarine-like stick products	9·2	2·2	5·0	11·0
Butter blend products	9·2	2·3	5·2	11·0
Butter, salted	11·5			
Butter, unsalted	11·5			
Whipped butter, salted	7·4			
Whipped butter, unsalted	7·4			
Total fat (% DV)				
Margarine/margarine-like tub and squeeze products	10	3	5	14
Margarine/margarine-like stick products	12	3	6	14
Butter blend products	12	3	7	14
Butter, salted	15			
Butter, unsalted	15			
Whipped butter, salted	9			
Whipped butter, unsalted	9			
SFA (g)				
Margarine/margarine-like tub and squeeze products	2·2	1·0	1·0	5·0
Margarine/margarine-like stick products	3·5	1·0	2·0	5·0
Butter blend products	3·5	1·1	2·0	6·0
Butter, salted	7·3			
Butter, unsalted	7·2			
Whipped butter, salted	4·3			
Whipped butter, unsalted	4·3			
SFA (% DV)				
Margarine/margarine-like tub and squeeze products	11	5	5	25
Margarine/margarine-like stick products	18	5	10	25
Butter blend products	18	6	10	30
Butter, salted	36			
Butter, unsalted	36			
Whipped butter, salted	21			
Whipped butter, unsalted	21			
MUFA (g)				
Margarine/margarine-like tub and squeeze products	2·8	1·6	1·0	6·0
Margarine/margarine-like stick products	3·1	1·2	1·5	5·3
Butter blend products	4·0	1·2	1·9	5·0
Butter, salted	3·0			
Butter, unsalted	3·3			
Whipped butter, salted	1·9			
Whipped butter, unsalted	1·9			
PUFA (g)				
Margarine/margarine-like tub and squeeze products	2·3	1·0	0·7	5·0
Margarine/margarine-like stick products	2·5	0·9	1·0	4·0
Butter blend products	1·2	0·5	0·5	2·0
Butter, salted	0·4			
Butter, unsalted	0·4			
Whipped butter, salted	0·3			
Whipped butter, unsalted	0·3			
*n*-3 (g)				
Margarine/margarine-like tub and squeeze products	0·4	0·2	0·0	0·9
Margarine/margarine-like stick products	0·3	0·1	0·2	0·5
Butter blend products	0·3	0·2	0·0	0·6
Butter, salted	0·0			
Butter, unsalted	0·0			
Whipped butter, salted	0·0			
Whipped butter, unsalted	0·0			
*n*-6 (g)				
Margarine/margarine-like tub and squeeze products	1·9	0·9	0·2	4·4
Margarine/margarine-like stick products	2·2	0·8	0·8	3·6
Butter blend products	0·8	0·3	0·4	1·3
Butter, salted	0·3			
Butter, unsalted	0·1			
Whipped butter, salted	0·2			
Whipped butter, unsalted	0·2			
Cholesterol (mg)				
Margarine/margarine-like tub and squeeze products	0·0	0·0	0·0	0·0
Margarine/margarine-like stick products	0·0	0·0	0·0	0·0
Butter blend products	12·8	5·9	1·7	25·1
Butter, salted	30·5			
Butter, unsalted	30·5			
Whipped butter, salted	21·2			
Whipped butter, unsalted	21·2			
Cholesterol (% DV)				
Margarine/margarine-like tub and squeeze products	0	0	0	0
Margarine/margarine-like stick products	0	0	0	0
Butter blend products	4	2	1	8
Butter, salted	10			
Butter, unsalted	10			
Whipped butter, salted	7			
Whipped butter, unsalted	7			

Min, minimum; Max, maximum; DV, daily value.

**Fig. 1:**
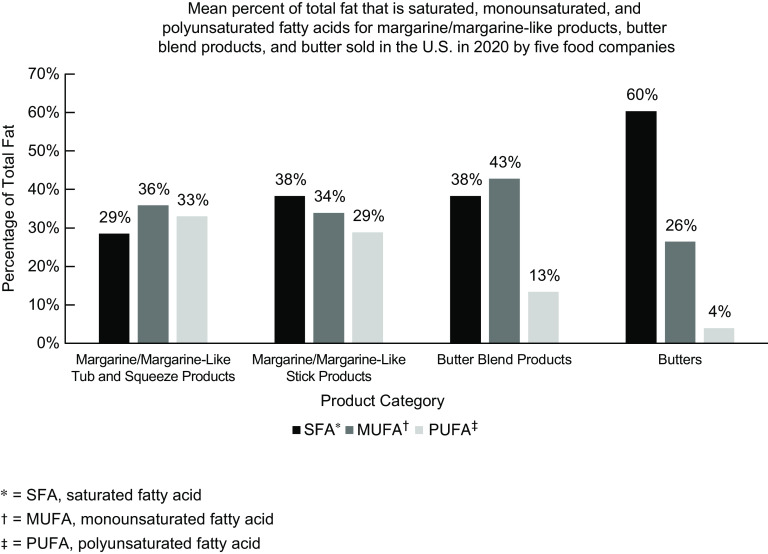
Mean percent of total fat that is SFA, MUFA and PUFA for margarine/margarine-like products, butter blend products and butter sold in the USA in 2020 by five food companies

Figure 1 shows the mean percent of total fat from SFA, PUFA and MUFA for each product category and for the combination of comparison butters. Margarine/margarine-like tub and squeeze products contained the lowest percent of SFA (29 %) and the highest percent of PUFA (33 %). In contrast, butter contained the highest percent of SFA (60 %) and lowest percent of PUFA (4 %).

*P*-values for the ANOVA tests carried out to examine differences in percent SFA, PUFA and MUFA by product type were statistically significant for SFA (*P* < 0·01) and PUFA (*P* < 0·01), but not for MUFA (*P* = 0·07).

The mean percent DV for selected vitamins and minerals are presented in Table 3. Mean sodium content across the margarine/margarine-like and butter blend product categories was 3 to 4 % DV, which was similar to that of regular salted butter (4 % DV). Mean vitamin A content for the margarine/margarine-like and butter blend categories ranged from 10 to 13 % DV. However, some products (*n* 7, 8 %) contained < 5 % DV for vitamin A. Mean vitamin E content ranged from 5 to 8 % DV for the margarine/margarine-like and butter blend categories. But, values ranged widely across products within the margarine/margarine-like product categories. For example, one of the products in the margarine/margarine like tub and squeeze category had 2 % DV while another contained 22 % DV for vitamin E.

**Table 3:** Percent daily value of sodium, vitamin A and vitamin E found in one tablespoon of margarine/margarine-like products, butter blend products and various butters sold in the USA in 2020 by five food companies

	Mean/value	sd	Min	Max
Sodium (% DV)				
Margarine/margarine-like tub and squeeze products	4	1	0	6
Margarine/margarine-like stick products	4	2	0	6
Butter blend products	3	1	1	4
Butter, salted	4			
Butter, unsalted	0			
Whipped butter, salted	2			
Whipped butter, unsalted	0			
Vitamin A (RAE) (% DV)				
Margarine/margarine-like tub and squeeze products	11	5	0	30
Margarine/margarine-like stick products	13	2	7	14
Butter blend products	10	4	3	14
Butter, salted	11			
Butter, unsalted	11			
Whipped butter, salted	7			
Whipped butter, unsalted	7			
Vitamin E (total *α*-tocopherol) (% DV)				
Margarine/margarine-like tub and squeeze products	7	5	2	22
Margarine/margarine-like stick products	8	3	4	12
Butter blend products	5	2	3	8
Butter, salted	2			
Butter, unsalted	2			
Whipped butter, salted	1			
Whipped butter, unsalted	1			

Min, minimum; Max, maximum; DV, daily value; RAE, retinol activity equivalents.

Calcium and vitamin D content were also examined. All comparison butters contained 0 % DV for these nutrients. Mean % DVs for these nutrients were also zero or low for all the margarine/margarine-like and butter blend product categories. However, maximum values indicated that some products were ‘good’ or ‘high’ sources of these nutrients, with values up to 10 % or 20 % DV.

## Discussion

Findings indicate that leading brands of margarine/margarine-like and butter blend products in the US marketplace are in compliance with the FDA mandate banning PHO. This is evidenced by the absence of any significant amount of *trans* fat in these products. While some products may retain naturally occurring *trans* fat, there is currently no set limit for consumption of naturally occurring *trans* fat. Current recommendations are to consume as little total *trans* fat as possible without diminishing the nutritional adequacy of the diet^(9)^, and the trace amounts that naturally occur in foods are not believed to contribute to important adverse outcomes^(20)^.

The margarine/margarine-like and butter blend products examined generally had fatty acid and energy profiles more consistent with current dietary recommendations^(9,21)^ than the butter comparisons. For all product categories, mean SFA content was at most half that of butter and mean PUFA content was at least double that of butter, including greater amounts of *n*-3 and *n*-6 fatty acids. The inclusion of *n*-3 fatty acids may be significant given the limited number of sources of *n*-3 fatty acids in the typical American diet^(22)^. Replacing SFA in the diet with unsaturated fat has been shown to have beneficial effects on total:HDL cholesterol ratios^(3)^ and to reduce serum total cholesterol and LDL-cholesterol^(23,24)^. Further evidence supports that replacing SFA with PUFA reduces CHD incidences^(25)^.

*The Dietary Guidelines for Americans 2020–2025* recommends that dietary cholesterol be consumed in as low amount as possible without compromising the nutritional quality of the diet^(9)^. All products contained less cholesterol than butter, with margarine/margarine-like products containing very small amounts (< 5 mg/1 tablespoon) or no cholesterol.

Between the product categories, margarine/margarine-like stick products and butter blend products contained similar fatty acid and energy profiles apart from small differences in MUFA and PUFA content. Margarine/margarine-like tub and squeeze products contained less saturated fat, total fat and energy than both margarine/margarine-like stick products and butter blend products. It is possible that the reduced energy content of these products may cause individuals to consume larger quantities, consequently causing the total energy and fat intake to be equal to or greater than that of other products or butter. However, based on findings for a one tablespoon amount, the energy and fatty acid profiles of margarine/margarine-like tub and squeeze products most align with current dietary guidelines. These products may also align more closely with current consumer interest in plant-based diets^(26)^ as these products likely contain minimal animal sources of fat given their lower SFA content relative to butter.

Findings suggest that consumers should continue to be advised to read the Nutrition Facts label on all individual products before purchasing as variations within product categories were found to occur. For example, the SFA content as a % DV ranged from 5 to 25 % across the margarine/margarine-like tub and squeeze products included in this study. However, it is important to note that products with the highest amount of SFA still contained less of this fatty acid than butter.

Average sodium content of all products was similar to that of regular-salted butter. Notably, the margarine/margarine-like product categories contained at least one sodium-free product, providing alternative options for individuals looking for sodium-free spreads.

Most products were ‘good’ sources of vitamin A, although a few products contained no vitamin A. Few products appeared to be fortified with calcium and vitamin D, both of which are nutrients not naturally occurring in butter. Given that these nutrients are generally under consumed in the American population^(27)^, public health could potentially benefit from more extensive fortification of these products with calcium and vitamin D.

Study findings show similarities and differences from previous studies. In 2003, the FDA published a final ruling stating *trans* fat must be labeled on all conventional foods and supplements^(28)^. In 2004, prior to the full implementation of this mandate, one study examined the *trans* fat content of products based on market share using GC and found all margarine products contained between 1·3 and 2·4 g *trans* fat per 14 g serving and contained amounts of SFA slightly less than most products in the present study^(6)^. A marketplace survey completed in 2006 after implementation of the mandate found that most margarines did not contain *trans* fat, but some margarines contained amounts between 0·5 and 2·5 g/serving, based on the serving size listed on the product’s Nutrition Facts label^(5)^. Products in this study were found to have similar amounts of total fat and slightly lower proportions of SFA than found in the present study.

Limitations of this study include non-random selection of butter blend and margarine/margarine-like products in the marketplace and reliance on expert judgement in identifying brands believed to be marketplace leaders. In addition, private label products available from leading food retailers, such as the Great Value brand from Walmart, are not included. As a result, findings should not be generalised beyond the specific products included in this evaluation. Another limitation is that chemical analysis was not used to determine the nutrient composition information for the products. Instead, formulations for the products were developed by NCC using information from the products’ Nutrition Facts label and ingredient list to estimate the nutritional content of the food. Therefore, limitations in the methods used by NCC to develop product formulations as well as inaccuracies and rounding on product labels may contribute to imprecise nutrient content values for products.

This study also has strengths. To our knowledge, this is the first study to examine the fatty acid profile of margarine/margarine-like products and butter blend products in the US marketplace post FDA ban on PHO. The study provides information on the full fatty acid profile, including fats not required on the Nutrition Facts label, such as MUFA, PUFA and *n*-3 and *n*-6 fatty acids. It also examines leading brand products, making the findings relevant to consumers in the USA.

## Conclusion

In the past, consumers have been advised to avoid margarine, especially hard or stick margarines, due to their *trans* fatty acid content^(8)^. However, study findings indicate that margarine/margarine-like and butter blend products in the US marketplace today have been reformulated post FDA ban on PHO and are a better choice than butter from a fatty acid content perspective. Findings also indicate that it remains wise to choose tub and squeeze margarine/margarine-like products over stick margarine/margarine-like or butter blend products.

Study findings may have implications for registered dietitian nutritionists, food manufacturers and public health professionals. Registered dietitian nutritionists should be advised to counsel clients on reading the Nutrition Facts label and provide education to the public on current formulations of margarine/margarine-like products and butter blend products. They should also leverage their training in food science to provide guidance on the use of spreads, as the altered fat content and composition of many spreads may change their properties in cooking and baking. Food manufacturers should be encouraged to continue to develop products high in unsaturated fatty acids and low in SFA, and consideration should be given to fortifying products with calcium and vitamin D. Finally, findings support the efficacy of public policy for public health promotion as the current findings demonstrate the FDA mandate for *trans* fat labeling and consecutive ban on PHO has successfully caused reformulation of products in the marketplace. Further research should examine the nutrient profiles of private label margarine/margarine-like products and butter blend products.

## Supplementary material

For supplementary material accompanying this paper visit https://doi.org/10.1017/S1368980021004511. click here to view supplementary material
